# Infant sex modifies associations between placental malaria and risk of malaria in infancy

**DOI:** 10.1186/s12936-020-03522-z

**Published:** 2020-12-03

**Authors:** Abel Kakuru, Michelle E. Roh, Richard Kajubi, Teddy Ochieng, John Ategeka, Harriet Ochokoru, Miriam Nakalembe, Tamara D. Clark, Theodore Ruel, Sarah G. Staedke, Daniel Chandramohan, Diane V. Havlir, Moses R. Kamya, Grant Dorsey, Prasanna Jagannathan

**Affiliations:** 1 London School of Hygiene and Tropical Medicine, London, UK; 2 Infectious Diseases Research Collaboration, 2C Nakasero Hill Road, Kampala, Uganda; 3 Department of Epidemiology and Biostatistics, University of California, San Francisco, USA; 4 Department of Obstetrics and Gynaecology, Makerere University College of Health Sciences, Kampala, Uganda; 5 Department of Medicine, University of California, San Francisco, USA; 6 Department of Pediatrics, University of California, San Francisco, USA; 7 School of Medicine, Makerere University College of Health Sciences, Kampala, Uganda; 8 Department of Medicine, Stanford University, Stanford, USA

**Keywords:** Placental malaria, Pregnancy, Infants, *Plasmodium falciparum*

## Abstract

**Background:**

Placental malaria (PM) has been associated with a higher risk of malaria during infancy. However, it is unclear whether this association is causal, and is modified by infant sex, and whether intermittent preventive treatment in pregnancy (IPTp) can reduce infant malaria by preventing PM.

**Methods:**

Data from a birth cohort of 656 infants born to HIV-uninfected mothers randomised to IPTp with dihydroartemisinin–piperaquine (DP) or Sulfadoxine–pyrimethamine (SP) was analysed. PM was categorized as no PM, active PM (presence of parasites), mild-moderate past PM (> 0–20% high powered fields [HPFs] with pigment), or severe past PM (> 20% HPFs with pigment). The association between PM and incidence of malaria in infants stratified by infant sex was examined. Causal mediation analysis was used to test whether IPTp can impact infant malaria incidence via preventing PM.

**Results:**

There were 1088 malaria episodes diagnosed among infants during 596.6 person years of follow-up. Compared to infants born to mothers with no PM, the incidence of malaria was higher among infants born to mothers with active PM (adjusted incidence rate ratio [aIRR] 1.30, 95% CI 1.00–1.71, p = 0.05) and those born to mothers with severe past PM (aIRR 1.28, 95% CI 0.89–1.83, p = 0.18), but the differences were not statistically significant. However, when stratifying by infant sex, compared to no PM, severe past PM was associated a higher malaria incidence in male (aIRR 2.17, 95% CI 1.45–3.25, p < 0.001), but not female infants (aIRR 0.74, 95% CI 0.46–1.20, p = 0.22). There were no significant associations between active PM or mild-moderate past PM and malaria incidence in male or female infants. Male infants born to mothers given IPTp with DP had significantly less malaria in infancy than males born to mothers given SP, and 89.7% of this effect was mediated through prevention of PM.

**Conclusion:**

PM may have more severe consequences for male infants, and interventions which reduce PM could mitigate these sex-specific adverse outcomes. More research is needed to better understand this sex-bias between PM and infant malaria risk.

*Trial registration* ClinicalTrials.gov, NCT02793622. Registered 8 June 2016, https://clinicaltrials.gov/ct2/show/NCT02793622

## Background

*Plasmodium falciparum* remains a major public health problem affecting mainly pregnant women and young children. In pregnant women infected with *P. falciparum*, parasitized erythrocytes sequester in the placenta, resulting in placental malaria (PM), which is characterized by placental inflammation, parasite infiltration, and deposition of malaria pigment, a product of digestion of haemoglobin by *P. falciparum* [1]. Placental malaria is associated with adverse effects such as preterm delivery, low birth weight, stillbirth and neonatal mortality [2–4]. Despite the use of preventive measures, including insecticide-treated nets and intermittent preventive therapy during pregnancy (IPTp), the burden of PM among pregnant women living in high malaria transmission settings remains high [5].

There is evidence that PM may impact infants after birth [6–8]. Several observational studies have reported associations between PM and increased risks of malaria, non-malaria febrile illnesses, and anaemia in infancy, possibly due to immune tolerance induced by in utero exposure to malaria antigens [9–13]. However, most of these studies defined PM as the detection of malaria parasites in placental blood by microscopy, which has limited sensitivity and does not account for past placental infections characterized by the presence of malaria pigment [14]. Furthermore, the severity of malaria pigment deposition in the placenta was recently shown to be strongly predictive of adverse birth outcomes including low birth weight and preterm birth [15]. Whether the severity of malaria pigment deposition in the placenta is also associated with the risk of malaria in infancy is unknown.

A recent double-blind randomised controlled trial compared the incidence of malaria during infancy among infants born to mothers who received monthly IPTp with dihydroartemisin-piperaquine (DP) *versus* Sulfadoxine–pyrimethamine (SP). In this trial, infants born to mothers receiving IPTp-DP had a lower malaria incidence compared to infants born to mothers receiving IPTp-SP. However, this association was observed in male, but not female, infants [16]. To further evaluate the association between PM and the incidence of malaria in infants, a secondary and mediation analysis of these data was carried out to examine how much of the previously observed associations between IPTp and the risk of malaria in infants is mediated through prevention of PM.

## Methods

### Study design, setting, and participants

Data were collected from a birth cohort of infants born to HIV-uninfected pregnant women enrolled in a randomised controlled trial of monthly IPTp with DP *vs* SP (Trial registration, ClinicalTrials.gov; NCT02793622) conducted in Busia district, Uganda, an area of perennial high malaria transmission intensity. Details of the study have been previously reported [5, 16, 17]. Pregnant women were enrolled at 12-20 weeks of gestation and followed through delivery. At delivery, placental blood and tissue samples were collected. Following delivery, all live births were followed up to 12 months of age. Mothers were encouraged to bring their infants to a dedicated study clinic open every day for all their medical care. Routine assessments were conducted every 4 weeks for clinical assessment and collection of blood smears for the detection of parasites by microscopy. Infants presenting with a history of fever in the past 24 h or a documented tympanic temperature ≥ 38.0 °C had a thick blood smear collected for detection of malaria parasites and those diagnosed with malaria were treated according to the Uganda Ministry of Health guidelines. Non-malarial illnesses were treated according to the integrated management of childhood illnesses guidelines. At 12, 28, and 52 weeks of age, blood was collected for haemoglobin measurement.

### Laboratory methods

Thick blood smears were stained with 2% Giemsa and read by microscopists [5]. Haemoglobin measurements were made using a spectrophotometer (Hemocue, Angelholm, Sweden). Malaria parasites were detected in placental blood by microscopy and loop-mediated isothermal amplification (LAMP) [18]. Placental biopsy specimens were embedded in paraffin wax, sectioned using a rotary microtome, fixed on glass slides, and dehydrated in sequential ethanol baths [19]. Separate slides were stained in 0.1% haematoxylin and 1% eosin for 5 and 1 min, respectively, or in 2% Giemsa for 30 min and examined for presence of intervillous parasite-infected erythrocytes and malaria pigment by two independent readers. The proportion of high-power fields (HPF) with malaria pigment deposition in fibrin was analysed as described [20].

### Study outcomes

The primary outcome was the incidence of malaria from birth to 12 months of age. An incident episode of malaria was defined as the presence of fever (history of fever in the past 24 h or a tympanic temperature ≥ 38·0 °C) with a positive thick blood smear not preceded by another malaria episode in the last 14 days. Secondary outcomes included time to first episode of malaria; incidence of complicated malaria (malaria with danger signs or meeting standardized criteria for severe malaria), all-cause hospitalizations; and non-malarial febrile illnesses; prevalence of malaria parasitaemia during routine visits and anaemia (haemoglobin < 10 g/dL); and infant mortality.

### Statistical methods

Data were double entered and verified in Microsoft Access, and statistical analyses conducted using Stata (14.2) including all live births with placental histology results. Follow-up began at birth and ended at 12 months of age or premature study withdrawal. The primary exposure variable, PM, was categorized as follows: no PM (absence of parasites or pigment); active PM (parasites detected in placental blood or tissue by microscopy LAMP or histology, with or without pigment [9, 21]); or past PM (presence of pigment, without parasites). Relationships between past PM and infant malaria incidence were initially evaluated by considering the proportion of HPF with pigment as a continuous variable. Given the non-linear nature of this relationship, past PM was further characterized into 2 groups based on best fit associations with the outcome variable: mild-moderate past PM (> 0–20% HPFs with pigment without parasites); or severe past PM (> 20% HPFs with pigment without parasites). Analyses were stratified by infant sex a priori. Associations between PM and the incidence of malaria were performed using negative binomial regression and adjusted for maternal parasitaemia status at enrollment, IPTp arm, gravidity, housing construction type, and clustering for twin gestation. The cumulative risk of any first episode of malaria was compared using a Cox proportional hazards model. For secondary outcomes, incident and repeated prevalence measures were compared using negative binomial regression model and generalized estimating equations with robust standard errors, respectively. Mediation analysis, using inverse odds weighting (IOW) [22], was used to estimate what proportion of the reported effect between maternal IPTp regimen and malaria incidence in infants [16] was mediated through preventing PM (Additional file 1). In brief, three models were used to conduct IOW mediation analyses. The first model used logistic regression to model treatment given mediator (PM) and mediator-outcome confounders. Predicted probabilities obtained from this model were then used to calculate treatment IOWs for each mother-infant pair. The second and third models used negative binomial regression to model the outcome given treatment with and without weights, respectively. The treatment coefficient from the model with weights estimated the direct effect, which was then subtracted from the treatment coefficient of the model without weights (total effect) to estimate the mediated effect. Bias-corrected 95% confidence intervals (CIs) were computed using bootstrapping. The proportion mediated by PM was calculated by dividing the mediated effect by the total effect. In all analyses, p-values of < 0·05 were considered statistically significant.

## Results

### Study profile and characteristics of study participants

**Fig. 1:**
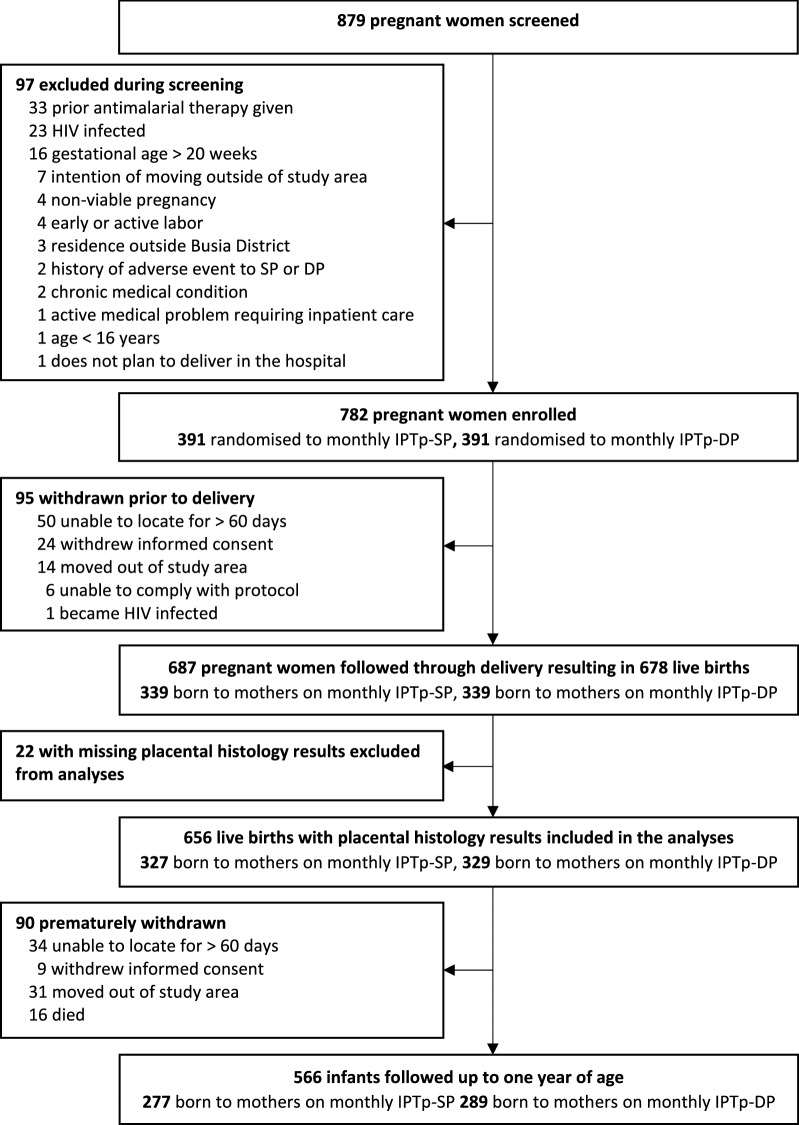
Study profile. *IPTp* intermittent preventive treatment in pregnancy, *DP* dihydroartemisinin–piperaquine, *SP* sulfadoxine–pyrimethamine

Between September 2016 and May 2017, 782 HIV-uninfected pregnant women were enrolled and randomised to receive either IPTp-SP or IPTp-DP, and 687 (87.9%) followed through delivery resulting in 678 live births (Fig. 1) [16]. A total of 656 infants with placental histology results were included in the analyses. Mean maternal age at enrollment was 24 years and 23.5% infants were born to primigravida mothers. During pregnancy, the incidence of malaria and prevalence of malaria parasitaemia were significantly lower among women randomised to IPTp-DP compared to those randomised to IPTp-SP. At delivery, women randomised to IPTp-DP had a significantly lower prevalence of active PM (2.1% versus 21.7% p < 0.001) and severe past PM (1.8% versus 12.2%, p < 0.001) compared to those randomised to IPTp-SP (Table 1).

**Table 1:** Characteristics of study participants

Characteristic	Maternal IPTp arm	
	Monthly SP (N = 327)	Monthly DP (N = 329)
Maternal characteristics at enrolment		
Age in years, mean (SD)	24.0 (5.9)	24.0 (5.7)
Gravidity, n (%)		
Primigravida/secundigravida	152 (46.5%)	156 (47.4%)
Multigravida	175 (53.5%)	173 (52.6)
House-hold type, n (%)		
Modern House	77 (23.6%)	71 (21.6%)
Traditional House	250 (76.5%)	258 (78.4%)
Parasite prevalence by microscopy or qPCR, n (%)		
No parasites	53 (16.2%)	63 (19.2%)
Sub-microscopic parasitaemia	111 (33.9%)	88 (26.8%)
Microscopic parasitaemia	163 (49.9%)	178 (54.1%)
Maternal characteristics during pregnancy		
Parasite prevalence by microscopy, n/N (%)^a^	797/2212 (36.0%)	355/2260 (15.7%)
Incidence of malaria (episodes/ppy)	0.59	0.09
Placental malaria status		
Placental malaria status, n (%)		
No PM	119 (36.4%)	232 (70.5%)
Active PM	71 (21.7%)	7 (2.1%)
Past PM (Mild-moderate pigment)	97 (29.7%)	84 (25.5%)
Past PM (Severe pigment)	40 (12.2%)	6 (1.8%)
Characteristics of infants at birth		
Preterm birth, n (%)	25 (7.7%)	17 (5.2%)
Gestation age in weeks, mean (SD)	39.4 (1.9)	39.6 (1.6)
Low birth weight, n (%)	33 (10.1%)	25 (7.6%)
Birth weight in grams, mean (SD)	3055 (505)	3024 (409)
Female sex, n (%)	161 (49.2%)	175 (53.2%)

*SP* Sulfadoxine–pyrimethamine, *DP* dihydroartemisinin–piperaquine, *SD* standard deviation, *ppy* per person year, *qPCR* quantitative polymerase chain reaction

^a^Defined as number of routine positive blood smears divided by total number of routine blood smears

*No PM* no parasites or pigment detected, *active PM* parasites detected with or without pigment, P*ast PM (Mild-moderate) * > 0–20% of high-power fields with pigment but no parasites, *Past PM (severe)* > 20%–60% of high-power fields with pigment but no parasites

### Association between placental malaria and the incidence of malaria in infants

**Fig. 2:**
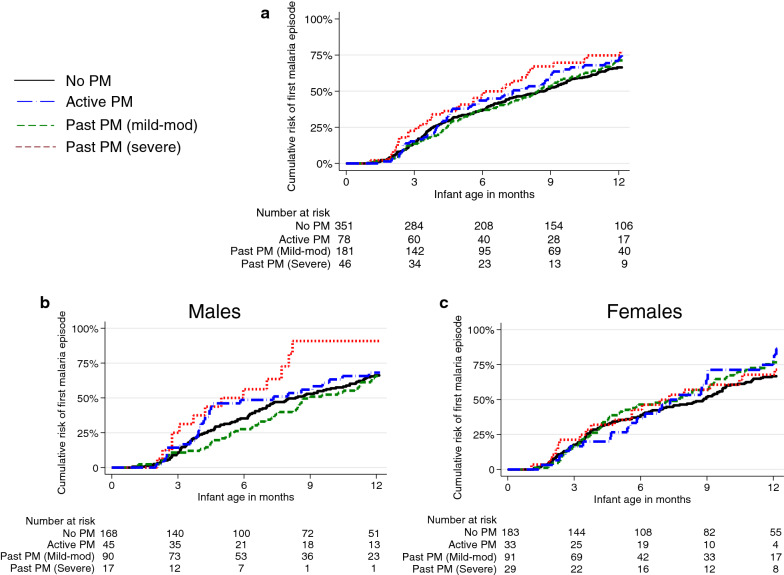
Time to first episode of malaria stratified by infant sex. **a** All infants, **b** male infants **c**  female infants. *PM* placental malaria, *mild-mod*  mild-moderate, *No PM* no parasites or pigment detected, *active PM* parasites detected with or without pigment, *past PM (mild-mod)* > 0–20% of high-power fields with pigment but no parasites, *past PM(severe)*  > 20%–60% of high-power fields with pigment but no parasites

Overall, 1088 incident episodes of malaria were diagnosed over 596.6 person years of follow-up (1.82 episodes per person year). Each 1% increase in the proportion of HPF with pigment deposition in fibrin was associated with a higher incidence of malaria in infants but the difference was not statistically significant (adjusted incidence rate ratio [aIRR] 1.98, 95% CI 0.76–5.20, p = 0.16). However, on stratifying by infant sex, significant interaction was observed (P-interaction [p_int_] = 0.03). Among male infants, a 1% increase in HPF with pigment deposition in fibrin was associated with over 5 times higher incidence of malaria (aIRR 5.20, 95% CI 1.70–15.94, p = 0.004). No significant difference in the incidence of malaria was observed in female infants (aIRR 0.53, 95% CI 0.13–2.11 p = 0.37). Considering PM as a categorical variable, compared to infants born to mothers with no PM, the incidence of malaria was higher among infants born to mothers with active PM (aIRR 1.30, 95% CI 1.00–1.71, p = 0.05) and those born to mothers with severe past PM (aIRR 1.28, 95% CI 0.89–1.83, p = 0.18, Table 2), but the differences were not statistically significant. However, effect modification by infant sex was also observed in the association between categories of PM and the incidence of malaria in infants (p_int_ = 0.02). Compared to no PM, severe past PM was associated with a higher incidence of malaria among male infants (aIRR 2.17, 95% CI 1.45–3.25, p < 0.001), but not among female infants (Table 2) .There were no significant associations between active PM or mild-moderate past PM and the incidence of malaria in male or female infants. There was no significant difference in the overall rate of first malaria episode among infants born to mothers with active PM (adjusted hazard ratio [aHR] 1.03, 95% CI 0.72–1.47, p = 0.88), mild-moderate past PM (aHR 0.92, 95% CI 0.71–1.18, p = 0.51), or severe past PM (aHR 1.18, 95% CI 0.75–1.86, p = 0.47), compared to those born to mothers with no PM. However, the association between PM and the rate of first malaria episode was also modified by infant sex. Compared to no PM, severe past PM was associated with a significantly higher rate of first malaria episode among male infants (aHR 1.99, 95% CI 1.04–3.81, p = 0.04; Fig. 2); no significant association was observed in female infants.

**Table 2:** Association between different measures of placental malaria and incidence of malaria during infancy

Infant category	Placental malaria categories (N)	Malaria episodes	Person years of follow-up	Malaria incidence^a^	Unadjusted		Adjusted^b^	
					IRR (95% CI)	*p* value	IRR (95% CI)	p-value
All	No PM (351)	574	327.4	1.75	Reference group		Reference group	
	Active PM (78)	152	68.9	2.21	1.27 (0.99–1.62)	0.06	1.30 (1.00-1.71)	0.05
	Past PM (mild-mod) (181)	271	157.8	1.72	0.97 (0.79–1.18)	0.74	0.94 (0.76-1.16)	0.55
	Past PM (severe) (46)	91	42.5	2.14	1.22 (0.91–1.63)	0.19	1.28 (0.89-1.83)	0.18
Male	No PM (168)	255	154.5	1.65	Reference group		Reference group	
	Active PM (45)	86	39.9	2.16	1.31 (0.93–1.87)	0.13	1.27 (0.87–1.84)	0.22
	Past PM (mild-mod) (70)	129	77.7	1.66	0.98 (0.72–1.35)	0.92	1.02 (0.76–1.36)	0.90
	Past PM (severe) (17)	49	15.3	3.20	1.87 (1.29–2.71)	0.001	2.17 (1.45–3.25)	<0.001
Female	No PM (183)	319	172.9	1.84	Reference group		Reference group	
	Active PM (33)	66	29.0	2.28	1.24 (0.89–1.73)	0.21	1.29 (0.87–1.91)	0.21
	Past PM (mild-mod) (91)	142	80.1	1.77	0.96 (0.73–1.24)	0.74	0.81 (0.61–1.08)	0.15
	Past PM (severe) (29)	42	27.1	1.55	0.85 (0.58–1.24)	0.40	0.74 (0.46–1.20)	0.22

*CI* confidence interval, *IRR* incidence rate ratio, *mild-mod* mild-moderate, *PM* placental malaria

^a^Episodes of malaria per person year of follow-up

^b^Adjusted for gravidity, maternal IPTp, maternal parasitaemia at enrolment, and household type

*No PM* no parasites or pigment detected, *active PM* parasites detected with or without pigment, *past PM (mild-mod)* > 0–20% of high-power fields with pigment but no parasites, *past PM(severe)* > 20%–60% of high-power fields with pigment but no parasites

### Association between placental malaria and other malaria outcomes in infants

The incidence of complicated malaria was non-significantly higher among infants born to mothers with active PM (aIRR 1.72, 95% CI 0.81–3.66, p = 0.16), and severe past PM (aIRR 2.44, 95% CI 0.93–6.37, p = 0.07; Table 3). The prevalence of parasitaemia detected during routine visits was also similar among infants born to mothers with active or past PM compared to infants born to mothers with no PM (Table 3). However, effect modification by infant sex was also observed in the association between PM and other malaria outcomes. Among male infants, severe past PM was associated with a nearly four-fold higher incidence of complicated malaria (aIRR 3.88, 95% CI 1.14–13.03, p = 0.03) and a more than two-fold higher prevalence of malaria parasitaemia during routine visits (risk ratio 2.24, 95% CI 1.34–3.74, p = 0.002) compared to no PM. No significant associations were observed among female infants (Table 3).

**Table 3:** Association between placental malaria and other malaria outcomes during infancy

Outcome measure	Infant category	Placental malaria categories	Number of cases (incidence PPY)	Unadjusted		Adjusted^a^	
				IRR (95% CI)	p-value	IRR (95% CI)	p-value
Incidence of complicated malaria	All	No PM (351)	30 (0.092)	Reference group		Reference group	
		Active PM (78)	12 (0.17)	1.89 (0.93–3.87)	0.08	1.72 (0.81–3.66)	0.16
		Past PM (mild-mod) (181)	14 (0.09)	0.97 (0.52–1.81)	0.91	0.99 (0.52–1.92)	0.98
		Past PM (severe) (46)	9 (0.21)	2.30 (0.97–5.43)	0.06	2.44 (0.93–6.37)	0.07
	Male	No PM (168)	13 (0.08)	Reference group		Reference group	
		Active PM (45)	6 (0.04)	1.78 (0.63–5.02)	0.27	1.18 (0.39–3.62)	0.77
		Past PM (mild-mod) (90)	11 (0.14)	1.68 (0.76–3.68)	0.20	1.54 (0.44–3.45)	0.30
		Past PM (severe) (17)	6 (0.39)	4.61 (1.50–14.22)	0.008	3.88 (1.14–13.03)	0.03
	Female	No PM (183)	17 (0.10)	Reference group		Reference group	
		Active PM (33)	6 (0.21)	2.09 (0.78–5.64)	0.15	2.60 (0.91–7.44)	0.14
		Past PM (mild-mod) (91)	3 (0.04)	0.38 (0.11–1.29)	0.12	0.44 (0.13–1.54)	0.20
		Past PM (severe) (29)	3 (0.11)	1.12 (0.34–3.66)	0.85	1.63 (0.46–5.81)	0.45

Prevalence measures	Infant category	Placental malaria categories	Prevalence n/N (%)	Risk ratio (95% CI)	p-value	Risk ratio (95% CI)	p-value
Prevalence of prasitaemia	All	No PM (351)	377/4170 (9.0%)	Reference group		Reference group	
		Active PM (78)	82/858 (9.6%)	1.07 (0.76–1.52)	0.70	1.19 (0.82–1.73)	0.36
		Past PM (mild-mod) (181)	154/1993 (7.7%)	0.85 (0.65–1.12)	0.26	0.86 (0.65–1.14)	0.28
		Past PM (severe) (46)	61/542 (11.3%)	1.22 (0.84–1.77)	0.30	1.38 (0.90–2.10)	0.14
	Male	No PM (168)	170/1964 (8.7%)	Reference group		Reference group	
		Active PM (45)	50/498 (10.0%)	1.17 (0.73–1.86)	0.52	1.19 (0.71–2.00)	0.51
		Past PM (mild-mod) (90)	70/976 (7.2%)	0.81 (0.55–1.20)	0.29	0.87 (0.58–1.31)	0.50
		Past PM (severe) (17)	31/191 (16.2%)	1.80 (1.08–2.99)	0.02	2.24 (1.34–3.74)	0.002
	Female	No PM (183)	207/2206 (9.4%)	Reference group		Reference group	
		Active PM (33)	32/360 (8.9%)	0.97 (0.57–1.66)	0.91	1.05 (0.61–1.83)	0.85
		Past PM (mild-mod) (91)	84/1017 (8.3%)	0.90 (0.61–1.31)	0.57	0.79 (0.53–1.17)	0.24
		Past PM (severe) (29)	31/351 (8.6%)	0.90 (0.54–1.51)	0.70	0.86 (0.48–1.52)	0.60

*CI* confidence interval, *IRR* incidence rate ratio, *mild-mod* mild-moderate, *PM* placental malaria, *PPY* per person year

^a^Adjusted for gravidity, maternal IPTp, maternal parasitaemia at enrolment, and household type

*No PM* no parasites or pigment detected, *active PM* parasites detected with or without pigment, *past PM (mild-mod)* = > 0–20% of high-power fields with pigment but no parasites; past PM(severe) = > 20%–60% of high-power fields with pigment but no parasites

### Association between placental malaria and non-malarial outcomes in infancy

There were 16 deaths (2.4% of infants) and 25 all-cause hospitalisations during follow-up. Severe past PM was associated with a non-significant higher incidence of all-cause hospitalisations among infants (aIRR 2.90, 95% CI 0.59–14.44, p = 0.19; Table 4) compared to no PM. There were no significant differences in the incidence of non-malaria febrile illnesses and prevalence of anaemia during routine visits among infants born to mothers in different PM categories compared to no PM (Table 4).

**Table 4:** Association between placental malaria and non-malaria outcomes in infants during the first year of life

Outcome measure	Placental malaria category (N)	Number of cases (incidence PPY)	Unadjusted		Adjusted^a^	
			IRR (95% CI)	p-value	IRR (95% CI)	p-value
Incidence of all-cause hospitalisations	No PM (351)	9 (0.027)	Reference group		Reference group	
	Active PM (78)	3 (0.044)	1.66 (0.40–7.00)	0.49	1.14 (0.22–5.98)	0.87
	Past PM (mild-mod) (181)	7 (0.044)	1.72 (0.58–5.12)	0.33	1.26 (0.44–3.64)	0.66
	Past PM (severe (46)	6 (0.141)	4.85 (1.38–17.00)	0.01	2.90 (0.59–14.44)	0.19
Incidence of non-malarial febrile illnesses	No PM (351)	1128 (3.45)	Reference group		Reference group	
	Active PM (78)	203 (2.95)	0.86 (0.72–1.03)	0.10	0.84 (0.64–1.10)	0.20
	Past PM (mild-mod) (181)	536 (3.40)	0.98 (0.86–1.13)	0.82	1.03 (0.84–1.25)	0.79
	Past PM (severe (46)	152 (3.58)	1.04 (0.85–1.26)	0.72	1.06 (0.72–1.57)	0.76

Prevalence measures	Categories	Prevalence n/N (%)	Risk ratio (95% CI)	p-value	Risk ratio (95% CI)	p-value
Anaemia^c^	No PM (351)	227/934 (24.3%)	Reference group		Reference group	
	Active PM (78)	48/190 (25.3%)	1.07 (0.76–1.50)	0.69	1.00 (0.69–1.47)	0.98
	Past PM (mild-mod) (181)	106/441 (24.0%)	1.01 (0.80–1.29)	0.92	0.94 (0.73–1.22)	0.64
	Past PM (severe (46)	37/118 (31.4%)	1.29 (0.94–1.77)	0.12	1.16 (0.79–1.70)	0.44

*CI* confidence interval, *IRR* incidence rate ratio, *mild-mod* mild-moderate, *PM* placental malaria

^a^Adjusted for gravidity, maternal IPTp, maternal parasitaemia at enrolment, and household type

^b^ Defined as haemoglobin < 10 g/dL measured routinely at 12, 28, and 52 weeks of age

*No PM* no parasites or pigment detected, *active PM* parasites detected with or without pigment, *past PM (mild-mod)* > 0–20% of high-power fields with pigment but no parasites, *past PM (severe)* > 20%–60% of high-power fields with pigment but no parasites

### Does prevention of PM explain the difference in infant malaria incidence between IPTp-DP and IPTp-SP?

Male infants born to mothers who received IPTp-DP have been previously reported to have a lower incidence of malaria in infancy compared to male infants whose mothers received IPTp-SP [16]. Consistent with this prior analysis, male infants with placental histology results born to mothers who received IPTp-DP had 23% less malaria than male infants born to mothers who received IPTp-SP (IRR 0.77, 95% CI 0.61–0.99, p = 0.049), but this association was not observed in female infants (Table 5). Mediation analysis showed that among all infants, 43% of IPTp-DP’s greater effect on preventing malaria during infancy than IPTp-SP was attributed to preventing PM (IRR_mediated_ 0.95, versus IRR_total_ 0.89, Table 5). In males, this proportion was 89.7% (IRR_mediated_ 0.79 *versus* IRR_total_ 0.77). Among female infants, the proportion of mediated effect was not calculated because the direct and mediated effects were in opposing directions.

**Table 5:** Effect of IPTp on malaria incidence in infants that is mediated by preventing placental malaria

Infant category	Total effect		Direct effect		Mediated effect		% of mediated effect^a^
	IRR (95% CI)	p-value	IRR (95% CI)	p-value	IRR (95% CI)	p-value	
All sexes	0.89 (0.75–1.04)	0.16	0.94 (0.73–1.17)	0.58	0.95 (0.77–1.16)	0.61	43.5%
Male	0.77 (0.61–0.99)	0.05	0.97 (0.67–1.36)	0.89	0.79 (0.56–1.09)	0.17	89.7%
Females	1.01 (0.81–1.26)	0.96	0.92 (0.71–1.22)	0.56	1.09 (0.87–1.38)	0.45	–

*CI* confidence intervals, *IRR* incidence rate ratio

IRRs represent the effect of IPTp DP versus SP on the incidence of malaria in infants. Confidence intervals reported here were obtained by bootstrapping and may differ from those reported in the primary analysis which used the delta method specifying robust standard errors

^a^Proportion of mediated effect was calculated using [ln(IRR_mediated effect_)/ln(IRR_total effect_)]*100

## Discussion

In this secondary analysis of data from a birth cohort of infants born to women randomised to receive monthly IPTp with SP vs DP, infants born to mothers with active PM and severe past PM had a non-significant higher incidence of malaria and complicated malaria during the first year of life compared to infants born to mothers without PM. However, the association between severe past PM and infant malaria was sex-specific. In male, but not female, infants, severe past PM was associated with a significantly higher incidence of malaria, a higher rate of first malaria episode, a higher incidence of complicated malaria, and a higher prevalence of parasitaemia during routine visits, compared to no PM. No sex-specific differences were observed between active PM and the incidence of malaria in infancy. Importantly, male infants born to mothers given IPTp-DP had significantly less malaria in infancy than males born to mothers given IPTp-SP, and 89.7% of this effect was mediated through prevention of PM.

Several prior studies have reported associations between active PM defined as detection of parasites in placental blood or tissue and an increased risk of malaria during infancy [9–11, 23–25]. Active PM detected by microscopy was associated with a higher risk of malaria infection in Ugandan infants [23], an increased rate of first parasitaemia in Beninese infants [11], and a higher risk of first episode of malaria in Gabonese infants [24]. In Mozambique, active PM detected by histology was also associated with higher odds of malaria compared to no PM [9]. The results from the current study, although not statistically significant, are consistent with these prior studies. The exact mechanisms through which PM might impact on the incidence of malaria in infancy are not well understood, but may be due to the effects of PM on the fetal immune system, including modulation of innate and adaptive cellular immune responses, as well as altered maternal–fetal transfer of antibodies to *P. falciparum* [26]. Alternatively, these associations may represent confounding secondary to shared levels of exposure to malaria parasites between mothers and their infants. To limit the effect of confounding by malaria exposure in the current study, IPTp arm and markers of exposure, including housing structure and maternal parasitaemia at enrolment were adjusted for, but the possibility of residual confounding persists.

Importantly, in this study, there was an association between the severity of past PM, defined as the proportion of HPF with malaria pigment deposition, with malaria risk, but only in male infants. Severity of malaria pigment deposition in the placenta has been previously reported as a strong predictor of adverse birth outcomes [15]. To our knowledge, this is the first report to suggest that severity of past PM is also associated with increased risk of malaria in infancy, and that infant sex may modify these associations. Although the precise mechanism by which infant sex modifies the relationship between PM and infant malaria risk remains uncertain, there is a growing body of evidence of sex-based differences in susceptibility to infectious diseases in infants [27, 28]. While one study in southern Sudan suggested that pregnant women who bore female infants were more likely to have PM than those who bore male infants [29], several adverse pregnancy outcomes, including stillbirths, have been shown to be more common in males than in females [30]. This suggests that in utero fetal exposures may have more severe consequences for male infants than female infants [31]. Furthermore, male infants exposed to malaria in utero have been shown to have higher frequencies of regulatory T cells in cord blood compared to female infants with similar exposure [32], suggesting that in utero malaria exposure may differentially induce tolerance to malaria antigens in male, but not female, infants. Alternatively, other sex-based differences, including malaria-induced responses to toll-like receptor ligands [27, 32], expression of x-chromosome encoded genes [33], and/or glucocorticoid receptor expression [34] may be responsible for these findings. Future studies are needed to better understand how PM may result in different sex-specific outcomes in infants.

IPTp with highly effective drugs such as DP can reduce the severity of PM, and in this study, IPTp-DP was associated with a statistically significant lower incidence of malaria among male infants, and a non-significant lower incidence of malaria among all infants, compared with IPTp-SP. In the mediation analysis, 89.7% of the effect of IPTp-DP vs SP on the incidence of malaria among male infants was mediated through prevention of PM. Although this result was not statistically significant, possibly due to limited sample size to conduct this stratified mediation analysis, these results suggest that in addition to preventing adverse birth outcomes in all infants, effective interventions in pregnancy that reduce severe PM may also result in a lower risk of malaria in male infants.

In this study, there was no significant association between PM and the incidence of non-malaria febrile illnesses in infancy, contrary to other reports that suggest that in utero exposure may also influence immune responses to non-malarial infections [7, 35]. Furthermore, effective prevention of PM with IPTp-DP was not associated with a lower incidence of non-malarial febrile illnesses in infancy compared with IPTp-SP [16]. These data suggest that PM may specifically impact infant malaria risk, although the possibility that it may impact other non-malaria infections cannot be excluded.

This study had some limitations. This secondary analysis was exploratory in nature and did not use categories of PM based on malaria pigment deposition in fibrin used by previous studies [15, 20]. However, prior studies assessed associations between the severity of placental pigment and adverse birth outcomes. This is the first study to assess associations between the severity of placental pigment deposition and infant malaria risk. It would be desirable to come up with a standardized classification system relating the severity of placental pigment deposition to infant malaria risk using data from multiple independent studies from different epidemiological settings. There were relatively small number of infants in the severe past and active PM categories, and these findings should, therefore, be interpreted with caution. The study was conducted in a very high malaria transmission setting and therefore its findings cannot be generalized to other malaria transmission settings. Finally, exclusion of infants with missing placental histology results (3.2%) and losses to follow-up may have reduced the power of the study and introduced bias if the infants excluded and those lost to follow-up were different from those who completed follow-up. However, there were no significant differences between infants excluded from the analysis, those lost to follow-up, and the infants who completed the study.

## Conclusions

Overall, this study suggests that severe malaria pigment deposition in placental tissue is associated with a higher incidence of malaria during the first year of life among infants residing in a setting of high malaria transmission intensity; however, this association was seen in only male infants. Highly effective interventions which reduce both severe past and active PM could be protective in male infants. Future research is needed to evaluate the association between PM and the risk of malaria in infancy in larger studies conducted in moderate and high malaria transmission settings and to evaluate mechanistic pathways between PM and infant malaria risk.

## Supplementary information

**Additional file 1.** Detailed description of mediation analysis using the inverse odds ratio weighting (IORW) approach.

## Data Availability

All data generated or analysed during the current study are available from the corresponding author on reasonable request.

## Abbreviations

aIRR: Adjusted incidence rate ratio
CI: Confidence interval
DP: Dihydroartemisinin–piperaquine
HPF: High-power fields
IOW: Inverse odds weighting
IPTp: Intermittent preventive therapy during pregnancy
IRR: Incidence rate ratio
LAMP: Loop mediated isothermal amplification
PM: Placental malaria
SP: Sulfadoxine–pyrimethamine
