# Supplementary material for: Infant sex modifies associations between placental malaria and risk of malaria in infancy
Source: Malar J. 2020 Dec 3;19:449. doi: 10.1186/s12936-020-03522-z (PMC7713316; doi:10.1186/s12936-020-03522-z)
Supplement: Supplementary file 1 — Additional file 1. Detailed description of mediation analysis using the inverse odds ratio weighting (IORW) approach. [file 12936_2020_3522_MOESM1_ESM.doc]

**Addition file: Appendix.** Detailed description of mediation analysis using the inverse odds ratio weighting (IORW) approach.

**Figure.** Directed acyclic graph depicting the relationship between mother’s IPTp regimen and infant malaria incidence mediated by mother’s malaria placental malaria infection status. Baseline confounders included maternal parasitaemia status at enrolment, gravidity, and a binary indicator of whether mother’s household was traditional versus modern. Given treatment randomization, we assumed there was no treatment-mediator and treatment-outcome confounding).

**Methods.** Mediation analyses were conducted using the inverse odds weighting (IOW) approach[1]. This approach applies treatment weights to outcome models to render treatment statistically independent from the mediator, thereby deactivating the indirect/mediated pathway (i.e. the effect of IPTp on infant malaria incidence that is mediated through preventing placental malaria; highlighted in blue in the above Figure). This deactivation then allows us to directly estimate the direct effect (i.e. the effect of IPTp on infant malaria incidence that is independent of placental malaria; highlighted above in red). The direct effect is then subtracted from the total effect to estimate the indirect (or mediated) effect. Compared to the traditional Baron and Kenny approach [2] of mediation analyses, main advantages of the IOW method are that it is non-parametric and can be used in presence of exposure-mediator interactions. However, IOW has been shown to produce larger standard errors than the traditional Baron Kenny approach [2].

Details on the methodology and Stata code are described in detail in Nguyen et al (2015) [1]. In brief, three models were specified. The first model used logistic regression to create treatment weights by specifying treatment (binary indicator of mother receiving DP) as a function of the mediator and potential mediator-outcome confounders (e.g. maternal parasitemia status at enrolment, gravidity and a binary indicator of whether mother’s household was traditional versus modern). Predicted probabilities were obtained from this model and used to create inverse odds as treatment weights for each mother-infant pair. For mother-infant pairs in the treated group (DP), weights were calculated by taking the inverse odds predicted from the first model: (1-predicted probability)/predicted probability. For mother-infant pairs in the control group (SP), treatment weights=1.

In the second model, we estimated the direct effect by using negative binomial regression to model the relationship between the outcome (infant malaria incidence) and treatment, weighted by the inverse odds from the first model (thus deactivating the indirect effect by making treatment and mediator statistically independent). The treatment coefficient in this model was then assumed to estimate the direct effect. The third model, we estimated the total/overall effect using negative binomial regression to model the relationship between the outcome and treatment *without* weights. The treatment coefficient in this model was then assumed to estimate the total effect, from which the direct effect (treatment coefficient in the second model) was subtracted to estimate the indirect effect.

Bootstrapping using 1,000 simulations were used to compute standard errors and bias-corrected 95% confidence intervals around effect estimates. The percent mediated by placental malaria was calculated using the following equation: [ln(IRR_indirect effect_)])/[ln(IRR_total effect_)]*100, where IRR=incidence rate ratio. All three models accounted for clustering of twin observations in the data by using vce(cluster ) option in Stata and negative binomial regression models incorporated infant days at-risk for malaria as an offset term.

**References**

1. Nguyen QC, Osypuk TL, Schmidt NM, Glymour MM, Tchetgen Tchetgen EJ. Practical guidance for conducting mediation analysis with multiple mediators using inverse odds ratio weighting. Am J Epidemiol **2015**; 181(5): 349-56.

2. Baron RM, Kenny DA. The moderator–mediator variable distinction in social psychological research: Conceptual, strategic, and statistical considerations. J Pers Soc Psychol **1986**; 51(6): 1173.
